# Prediction models for post-thrombectomy brain edema in patients with acute ischemic stroke: a systematic review and meta-analysis

**DOI:** 10.3389/fneur.2023.1254090

**Published:** 2023-08-31

**Authors:** Lei Liu, Chun-yu He, Jia-xin Yang, Si-ting Zheng, Jun Zhou, Ye Kong, Wen-bo Chen, Yan Xie

**Affiliations:** School of Nursing, Chengdu Medical College, Chengdu, Sichuan, China

**Keywords:** acute ischemic stroke, thrombectomy, brain edema, prediction model, systematic review

## Abstract

**Objective:**

The objective of this study is to systematically evaluate prediction models for post-thrombectomy brain edema in acute ischemic stroke (AIS) patients. This analysis aims to equip clinicians with evidence-based guidance for the selection of appropriate prediction models, thereby facilitating the early identification of patients at risk of developing brain edema post-surgery.

**Methods:**

A comprehensive literature search was conducted across multiple databases, including PubMed, Web of Science, Embase, The Cochrane Library, CNKI, Wanfang, and Vip, aiming to identify studies on prediction models for post-thrombectomy brain edema in AIS patients up to January 2023. Reference lists of relevant articles were also inspected. Two reviewers independently screened the literature and extracted data. The Prediction Model Risk of Bias Assessment Tool (PROBAST) and the Transparent Reporting of a Multivariable Prediction Model for Individual Prognosis or Diagnosis (TRIPOD) guidelines were employed to assess study bias and literature quality, respectively. We then used random-effects bivariate meta-analysis models to summarize the studies.

**Results:**

The review included five articles, yielding 10 models. These models exhibited a relatively high risk of bias. Random effects model demonstrated that the AUC was 0.858 (95% CI 0.817–0.899).

**Conclusion:**

Despite the promising discriminative ability shown by studies on prediction models for post-thrombectomy brain edema in AIS patients, concerns related to a high risk of bias and limited external validation remain. Future research should prioritize the external validation and optimization of these models. There is an urgent need for large-scale, multicenter studies to develop robust, user-friendly models for real-world clinical application.

**Systematic review registration:**

https://www.crd.york.ac.uk, unique Identifier: CRD42022382790.

## 1. Introduction

AIS presents a substantial healthcare challenge due to its high incidence and disability rate (1). Recent clinical guidelines highlight the importance of endovascular mechanical thrombectomy (EVT) as an effective early treatment strategy for AIS (2). However, even with successful reperfusion achieved via early endovascular thrombectomy, approximately 50% of patients with AIS still encounter a range of adverse outcomes, including mortality (3). One significant complication contributing to early death in these patients is brain edema post-thrombectomy, with occurrence rates ranging from 10 to 75% (4). This condition often results in malignant neurological deterioration accompanied by significant brain swelling, potentially leading to tonsillar herniation, death, or functional impairment (5). Current therapeutic guidelines suggest decompressive craniectomy within 48 h of onset as an effective treatment for brain edema (6). Studies have shown a statistically significant difference in adverse outcomes between patients who underwent decompressive craniectomy within this 48-h window and those who did not (7). This highlights the necessity of early identification of high-risk patients and timely intervention to prevent or reduce the incidence of post-thrombectomy brain edema. Several researchers have aimed to create and validate predictive models to estimate the risk of post-thrombectomy brain edema in AIS patients. These models are intended to enhance early intervention and implement stratified management, thereby improving patient recovery. Despite these efforts, there is considerable variation among these studies in terms of study population, modeling methods, follow-up duration, and outcome measures. This systematic review aims to critically assess these models to inform their construction and application, as well as contribute to clinical strategies for preventing brain edema in AIS patients after thrombectomy. By conducting this review, we aspire to fill the current knowledge gaps and provide valuable insights to guide future research and improve clinical practice.

## 2. Methods

This systematic review has been registered with PROSPERO (ID: CRD42022382790) and was conducted following the PRISMA guidelines.

### 2.1. Literature search strategy

We conducted an exhaustive and well-structured literature search across multiple databases, including PubMed, Web of Science, Embase, The Cochrane Library, CNKI, Wanfang, and Vip. Our goal was to meticulously identify relevant studies that focus on the development and validation of prediction models for brain edema in AIS patients post-thrombectomy. The search range spanned from the inception of each individual database up to January 2023. We further fortified our search process by manually scouring through the reference lists of the studies initially identified, to ensure no pertinent study was overlooked. The search was executed using carefully selected terms, which include: “Ischemic Stroke,” “Brain Ischemia,” “Brain Edema,” “Cerebral Edema,” “mechanical thrombectomy,” “endovascular thrombectomy,” and “risk prediction model.” Supplementary Table S1 for detailed search strategy.

### 2.2. Inclusion and exclusion criteria

Inclusion Criteria: (i) Study Types: We considered both case–control and cohort studies; (ii) Study Participants: The studies needed to involve patients aged 18 years or older diagnosed with stroke, confirmed by CT or MRI, based on widely accepted national and international diagnostic and classification criteria; (iii) Study Focus: We included studies that developed and/or validated prediction models for post-thrombectomy brain edema risk. Malignant edema was required to be defined as a syndrome of clinical deterioration (or death or the need for decompressive surgery) accompanied by imaging evidence of brain swelling. The studies must have measured at least one potential predictor or a predictive model for the development of malignant edema.

Exclusion Criteria: (i) We excluded prediction models that included non-large vessel occlusion (LVO) stroke patients, such as those with intracerebral hemorrhage or lacunar stroke; (ii) Duplicate publications or studies deriving from the same cohort were not considered; (iii) Studies with incomplete or insufficient reporting of model development information or other pertinent details (e.g., absence of model performance evaluation) were excluded. Owing to limited information, conference abstracts, review articles, letters, comments, editorials, and errata were not included in the analysis.

### 2.3. Literature selection and data extraction

Two independent researchers were tasked with screening the literature based on the pre-established inclusion and exclusion criteria. Subsequent to this initial screening, data extraction was carried out utilizing a standardized form that was formulated based on the critical appraisal and data extraction for systematic reviews of prediction modelling studies (CHARMS) (8). This detailed extraction involved collecting specific information from each study, such as the first author’s name, publication year, title, study’s country of origin, study type, sample size, data source, AIS diagnostic method, the number of developed models, outcome measures, candidate variables, modelling techniques, variable selection methods, model performance, validation methods, model presentation format, the number and names of predictive factors, and the method used for managing missing values. Meanwhile, we conducted a comparative analysis of the five literatures, focusing on the inclusion criteria, target population, and diagnostic criteria for cerebral edema in each study. We organized this information into a table to facilitate comparison. Supplementary Table S2 for detailed information.

### 2.4. Risk of bias and quality evaluation

The quality of the included studies was assessed using the Transparent Reporting of a Multivariable Prediction Model for Individual Prognosis or Diagnosis (TRIPOD) guidelines (9). These guidelines provide a framework for transparent reporting of multivariable prediction models for individual prognosis or diagnosis. Additionally, the Prediction Model Risk of Bias Assessment Tool (PROBAST) (10) was employed to evaluate the risk of bias and applicability of the included studies in the context of prediction model research. Two researchers independently carried out these assessments and cross-verified the results. In instances of disagreement where consensus could not be reached through discussion, the opinion of a third party was solicited to resolve the issue.

### 2.5. Data analysis methods

Model performance was quantified by the area under the subject operating characteristic curve (AUC). In this meta-analysis, we extracted the AUC and 95% confidence interval (CI) of the relevant model and calculated the standard error of the AUC values using the formula provided by Bradley et al. (11). In addition, the results of the five included studies were quantitatively summarized and analyzed. The meta analyses were conducted using the MedCalc Statistical Software (version 22.007).

## 3. Results

### 3.1. Literature search results

**Figure 1:**
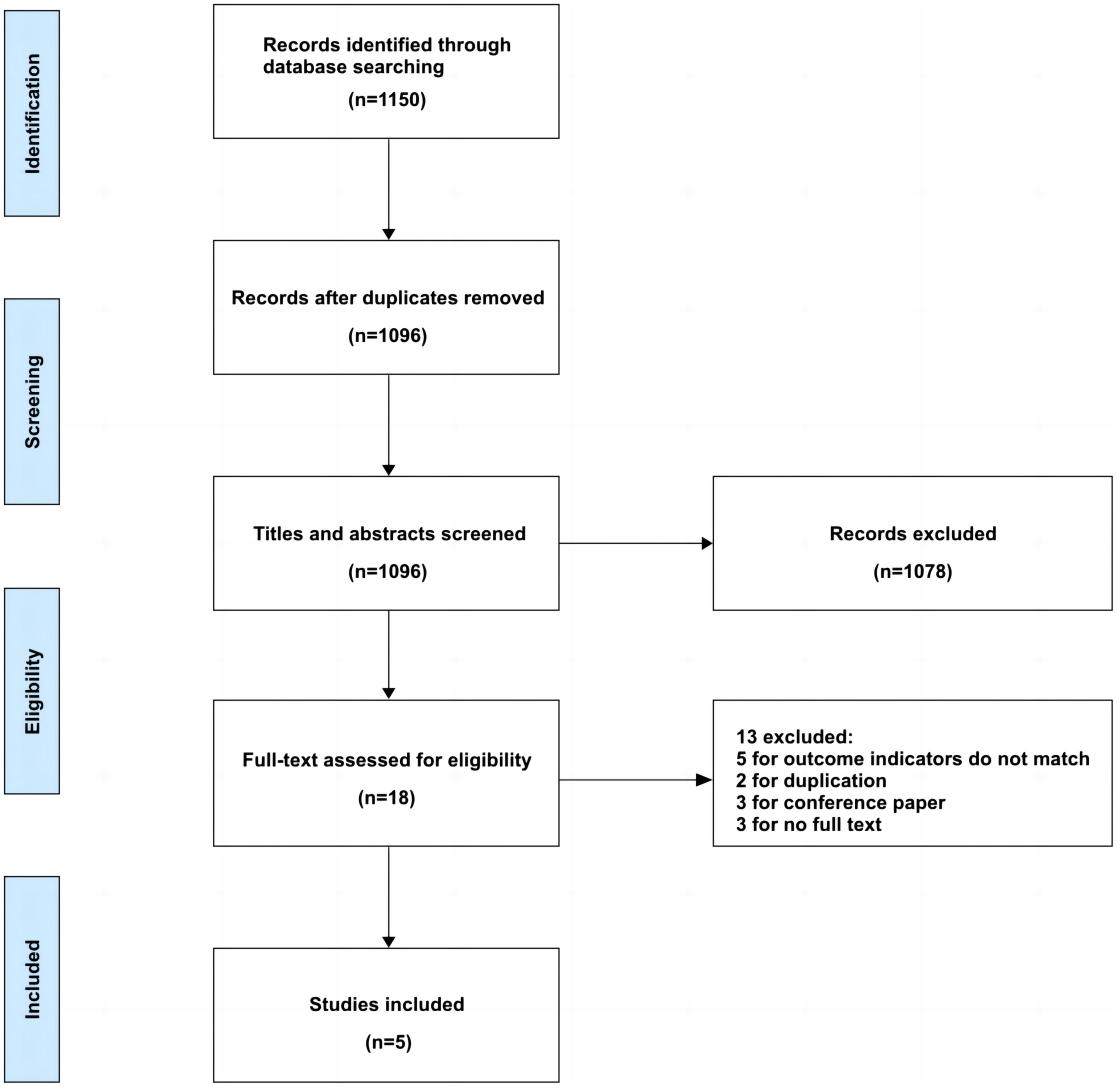
Flow chart of literature search.

Our preliminary search identified 1,150 articles. After removing duplicates, 1,096 articles remained for screening. Following the predetermined inclusion and exclusion criteria, we reviewed the titles, abstracts, and full texts of these articles. Ultimately, we included 5 articles, representing 10 brain edema risk prediction models. The detailed flowchart of the literature screening process is provided in Figure 1.

### 3.2. Basic characteristics of the included literature

We included a total of five articles in this review (12–16), all of which were retrospective cohort studies. Four of these articles were published in English (12–15), and one was published in Chinese (16). One study was multicenter in design (13), while the remaining four were conducted in single-center settings (12–14, 16). Four studies included both preoperative and postoperative predictive variables, while one study included only preoperative factors (16). The studies included in our review offer a range of models for predicting post-thrombectomy brain edema in AIS patients. For instance, the study by Chen (12) uses a logistic regression model, identifying 24-h CT ASPECT scores, cisternal effacement, hypertension, and complete recanalization as key predictive factors. On the other hand, the study by Zeng (15) explores multiple machine learning models, focusing on hypodensity volume and proportion and TOAST-LAA as important features. This diverse array of models and identified variables underscore the complexity of predicting post-thrombectomy brain edema in AIS patients and indicate the need for further research in this area. The basic characteristics of the included articles are summarized in Table 1.

**Table 1:** Characteristics of the included studies.

Study	Year	Model	Model numbers	Imaging modality	Sample size	Sample size of outcome events	Variable filtering	Important feature identified
Chen (12)	2019	LR	1	NCCT	199	87	Univariate analysis	24-h CT ASPECT scores, cisternal effacement, hypertension and complete recanalization
Du (13)	2020	LR	1	CT	370	71	Univariate analysis	Age, baseline NIHSS score, collateral circulation, fast blood glucose level and recanalization
Jiang (14)	2022	LR	1	CT	329	72	LASSO	Basal cistern effacement, postoperative NIHSS score, brain atrophy, hypoattenuation area and stroke etiology
Zeng (15)	2022	SVW, RF, XGBoost, KNN, GBN	5	NCCT	110	34	Univariate analysis	Hypodensity volume and proportion、TOAST-LAA
Cheng (16)	2022	LR, XGBoost	2	CT, MRI	382	41	XGBoost algorithm model	LR:age, admission NIHSS score, grade of collateral circulation, number of thrombus removal, time from onset to vascular recanalization
								XGBoost:grade of collateral circulation, number of thrombectomy, time from onset to vascular recanalization, admission NIHSS score, age

LR, logistic regression; NCCT, Non-Contrast CT; ASPECTS, Alberta Stroke Program Early CT Score; SVW, support vector machine; RF, random forest; XGBoost, extreme gradient boosting; KNN, K-Nearest Neighbor; GBN, Gaussian Bayesian network; NIHSS, National Institute of Health stroke scale.

### 3.3. Model performance and validation

**Figure 2:**
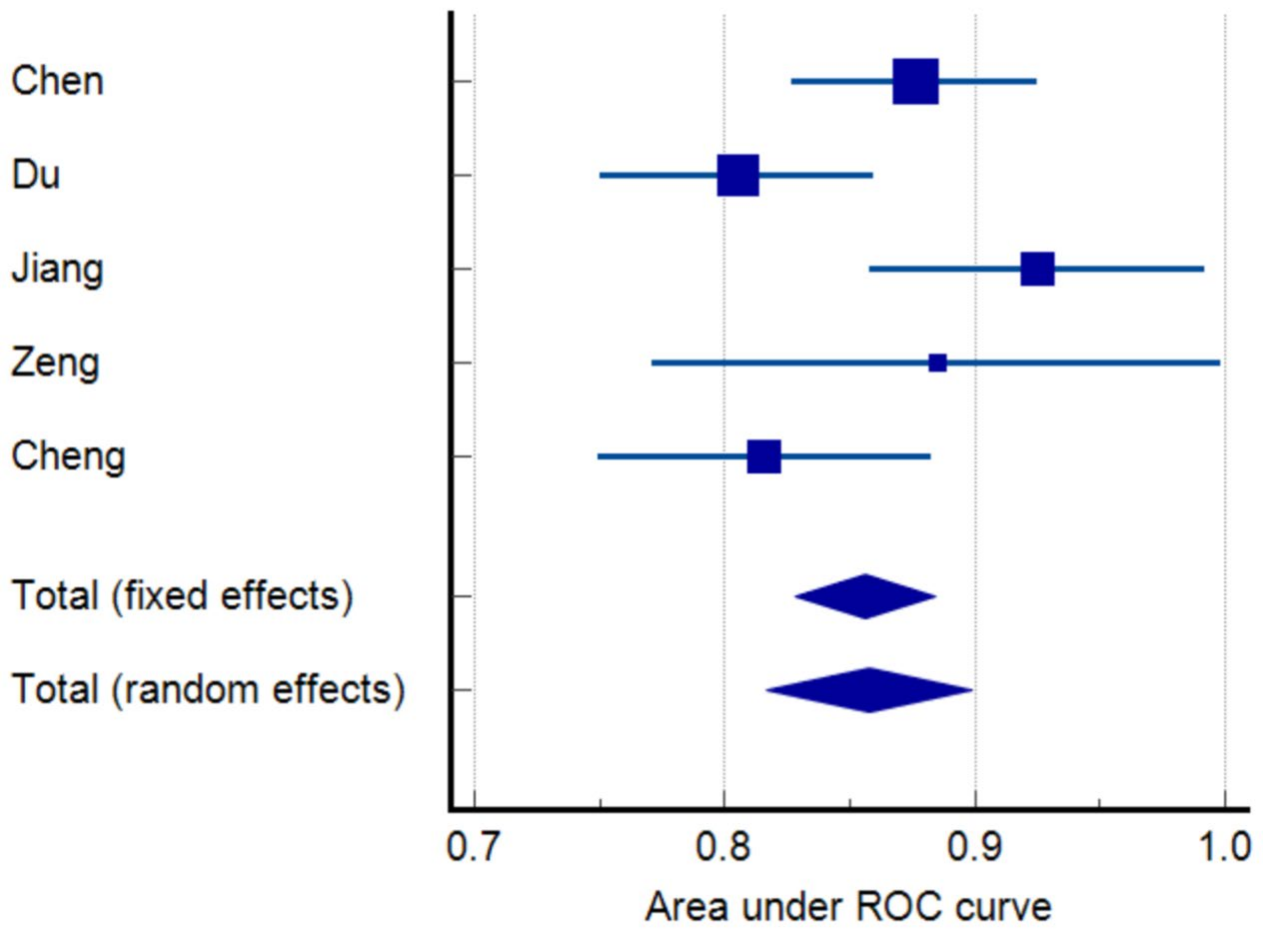
Meta-analysis of the area under the receiver-operating characteristics (ROC) curves (AUC) of models predicting functional outcome.

When constructing a model, certain metrics such as AUC can provide valuable insights about the model’s performance. The five studies included in our analysis reported AUC values ranging from 0.805 to 0.925. Random effects model demonstrated that the AUC was 0.858 (95% CI 0.817–0.899). Furthermore, in terms of model calibration, four papers reported calibration methods (12–14, 16), all of which were Hosmer–Lemeshow goodness-of-fit tests, and three of these studies also employed calibration graphs for further evaluation (12–14). Additionally, two studies performed decision curve analysis (DCA) (14, 15). Beyond the AUC, we examined several performance metrics such as sensitivity, specificity, and accuracy. AUC is used to assess the model’s ability to discriminate between positive and negative cases, whereas sensitivity, specificity, and accuracy provide more specific information about the model’s performance, as shown in Table 2. For example, the model of Jiang (14) has a sensitivity of 69.4%, a specificity of 93.0%, a positive predictive value of 73.5%, and a negative predictive value of 91.6%, while the model of Zeng (15) has a sensitivity of 0.900, a specificity of 0.913, and an accuracy of 0.909. These additional performance metrics provide us with richer information and help us to more comprehensive understanding of the model’s performance. However, it is important to note that since not all studies reported these additional performance metrics, we were unable to analyze them together. In terms of model verification, all the studies carried out internal verification, but Cheng did not specify their internal verification methods (16). Models are represented in different formats, including equations and graphs. Further details are presented in Figure 2 and Table 2.

**Table 2:** Model performance and validation.

Study	Model	AUC	Calibration method	Additional performance metrics	Internal validation	Model visualization
Chen (12)	LR	0.876 (0.822, 0.918)	Hosmer–Lemeshow: *p* = 414	–	Bootstrap	Nomogram
Du (13)	LR	0.805 (0.750, 0.860)	Hosmer–Lemeshow: *p* = 0.681	–	Bootstrap	Nomogram
Jiang (14)	LR	0.925 (0.890, 0.961)	Hosmer–Lemeshow: *p* = 0.386	SEN:69.4% SPE:93.0% PPV:73.5% NPV:91.6%	Nomogram	Web-based dynamic nomogram
Zeng (15)	LR stacking	0.885 (0.738, 1.000)	–	SEN:0.900 (0.555, 0.998) SPE:0.913 (0.720, 0.989) ACC:0.909 (0.757, 0.981)	Web-based dynamic nomogram	–
Cheng (16)	LR	0.816 (0.749, 0.883)	Hosmer–Lemeshow: *p* = 0.438	–	–	–

### 3.4. Risk of bias assessment

**Figure 3:**
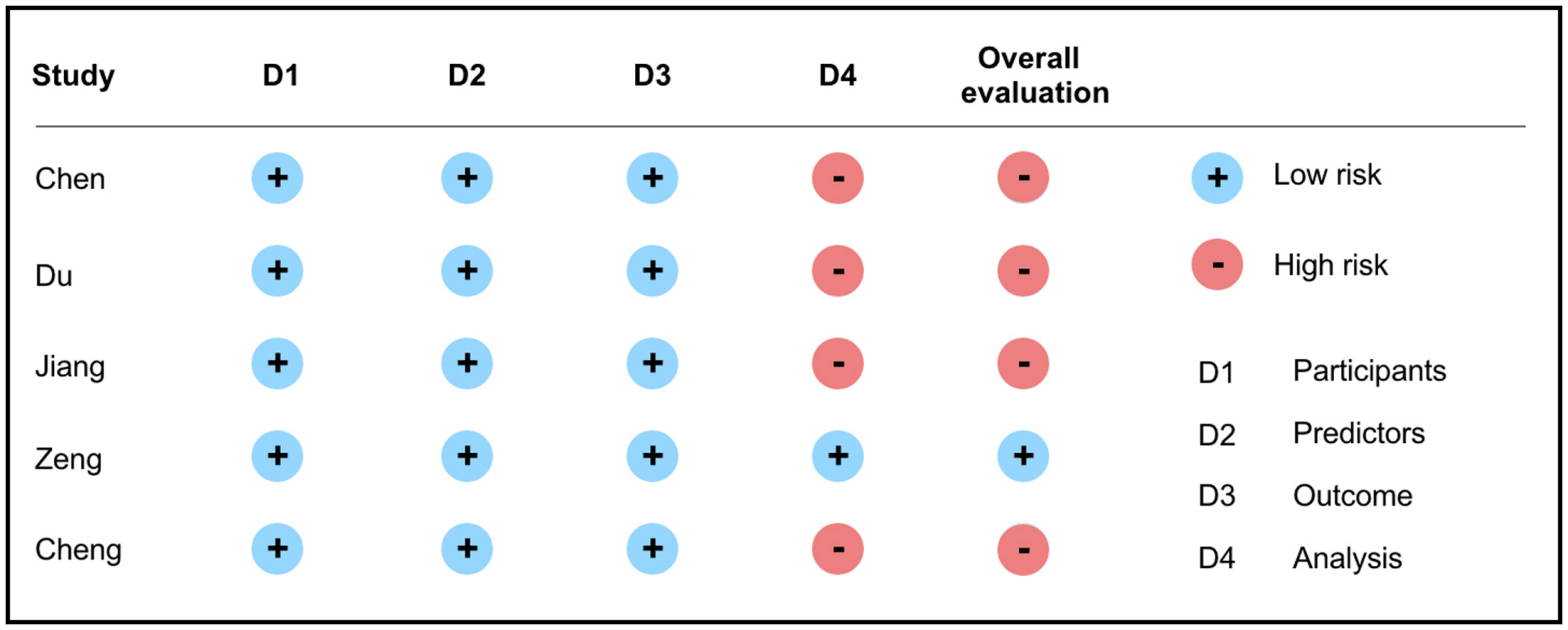
Results of bias risk and applicability evaluation of the included literature.

Bias risk assessment was carried out using the PROBAST tool. The results showed that among the five included articles, only one presented a low risk of bias (15), while the remaining four had issues primarily within the analysis domain. These issues encompassed inappropriate handling of continuous or categorical variables, improper management of missing data, variable selection based solely on univariable analysis, and inadequate handling of complex issues in the data (12–14, 16). Further details are presented in Figure 3.

### 3.5. Quality assessment of the literature

Our quality assessment, conducted based on the Transparent Reporting of a multivariable prediction model for Individual Prognosis Or Diagnosis (TRIPOD) guidelines, indicates that the overall quality of the studies included in this review is quite high, covering more than 70% of the reporting items. However, we also noticed certain limitations or lack of clarity in reporting certain aspects. For example, despite Chen’s study (12) being comprehensive, it lacks detailed documentation of the sample size calculation process and clear guidelines on the use of the predictive model. Similarly, Du’s study (13) is also thorough but falls short in providing sufficient details about the parameters of their predictive model and its application. Jiang’s study (14) did not specify the method for calculating the sample size nor explain the parameters of their predictive model in their detailed report. Lastly, although Cheng’s study (16) is methodologically extensive, it did not specify how they determined the sample size, the specific parameters of their model, and how to apply their model in practice. Notwithstanding the TRIPOD guidelines providing us with an authoritative tool for assessing the quality of predictive models, we recognize that due to the models’ complexity and diversity, this evaluation may not fully capture all the details crucial for predictive modeling. For instance, sample size significantly affects the model’s robustness and accuracy, while the selection and adjustment of model parameters are essential for model optimization and predictive capability. Supplementary Table S3 for detailed quality assessment.

## 4. Discussion

### 4.1. Overall performance of risk prediction models for post-thrombectomy brain edema in stroke patients

Through a comprehensive search and selection process, we identified five original studies (12–16) focusing on the predictive factors and models for malignant brain edema following ischemic stroke. These studies, all of which involved model development, demonstrated robust performance, with AUC values ranging from 0.805 to 0.925, all surpassing the 0.8 threshold. This indicates strong predictive capability of these models for post-thrombectomy brain edema. Notably, all 10 models constructed across these five studies underwent internal validation. This step is crucial in assessing the model’s predictive power for future data, detecting overfitting, enhancing prediction accuracy, and ensuring the reliability and value of the results (17) In addition, most of the included studies presented their model outcomes in the form of nomograms. As graphical representations of predictive statistical models, nomograms offer visualization advantages and ease of use, enabling intuitive, convenient, and effective individualized risk prediction, thereby facilitating their clinical application by healthcare professionals (18). Of particular note, Jiang et al. (14) presented their final model using an online dynamic nomogram, which allows healthcare professionals to directly input patient-specific information and obtain accurate prediction results.

### 4.2. Identifying effective predictive factors for post-thrombectomy brain edema in stroke patients

We generalized the predictor variables of the included prediction models and found that these predictors could be broadly classified into the following categories: age, NIHSS score, successful recanalization, cerebral blood supply, presence of hypertension, area of cerebral ischemia, and etiology of stroke, which did not appear in all models, possibly due to study design, sample characteristics, extent of disease, and other clinical variable Differences. Based on the results of the available studies, age, NIHSS score, successful recanalization, and cerebral collateral circulation grading are by far the most frequently cited valid predictors of cerebral edema.

Research focusing on stroke patients indicated that the risk of post-thrombectomy brain edema significantly escalates for patients over 80 years of age (19). This finding could be attributed to the reduced elasticity and impaired perfusion in brain tissue of the elderly, which may heighten the likelihood of brain edema following thrombectomy. Furthermore, the diminished vascular elasticity and compromised blood circulation in the elderly can facilitate local thrombus formation, thereby exacerbating brain edema symptoms (20). This finding could be attributed to the reduced elasticity and impaired perfusion in brain tissue of the elderly, which may heighten the likelihood of brain edema following thrombectomy. Furthermore, the diminished vascular elasticity and compromised blood circulation in the elderly can facilitate local thrombus formation, thereby exacerbating brain edema symptoms (21). Successful reperfusion serves as a protective factor against post-thrombectomy brain edema in stroke patients (22). Restoring blood perfusion post-reperfusion can enhance cerebral blood flow, mitigate brain ischemia and hypoxia, restore normal metabolic function, and minimize inflammatory responses and tissue damage (23). This process consequently reduces the brain edema incidence, highlighting the importance of preoperative evaluation of thrombectomy indications and risks in stroke patients, as well as close postoperative patient monitoring. Collateral circulation signifies the compensatory blood supply from peripheral collateral arteries when the brain’s main arteries are occluded, thereby maintaining normal cerebral metabolic function (24). However, thrombectomy can lead to a sudden surge in cerebral perfusion due to rapid blood flow restoration following thrombus removal. This abrupt change can exacerbate insufficient collateral circulation and contribute to brain edema (25). Thus, postoperative assessment and evaluation of collateral circulation status are necessary, encompassing the distribution, quality, and quantity of collateral circulation, to guide appropriate treatment measures. However, ensuring the prioritization of predictor variables is currently a challenge due to the differences in predictor variables between different predictive models. In Zeng’s study (15), Shapley Additive Explanation (SHAP) was used to prioritize the variables, which is a game-theoretic based method that fairly assigns predictive values to each of the feature’s contribution, which provides an effective way to interpret the predictor variables of our model. Future research should further adopt this approach to explore effective predictors to improve the accuracy of predictive models.

### 4.3. Limitations in predictive models for post-thrombectomy brain edema risk in stroke patients

Based on our literature search and screening, the existing predictive models for post-thrombectomy brain edema risk in stroke patients remain in their developmental stages. A majority of the studies included in this review demonstrated a high risk of bias in their assessments, primarily in the analytical domain. Moreover, several limitations in methodological quality and reporting exist, which we summarize as follows: First, regarding the geographic distribution of the included studies, all of the prediction models for the risk of post-thrombectomy brain edema were developed based on Chinese populations and most of them were single-center studies, which has some limitations in terms of the target population of the model prediction. Although these studies have demonstrated strong predictive power, this may affect the generalizability of the models. Multi-center, multi-country studies covering more regions and a wider range of populations tend to have results that are more representative and generalizable, thus enabling the predictive models to be applied to a wider range of patient populations. Secondly, shortcomings were observed in data analysis and processing methods. Primarily, most studies did not perform pre-processing on the data, especially regarding missing data and the handling of continuous or categorical variables. None of the five included studies reported on this, potentially compromising the quality of the data. This could result in biased prediction outcomes and a decrease in model performance (26), thereby impacting the accuracy in real-world application. Meanwhile, none of the five studies in this review reported on the treatment of missing data. Failing to handle missing values and directly excluding them could lead to bias in both the prediction outcomes and model performance. Furthermore, only Zeng addressed the issue of category imbalance (15). They employed a data sampling technique known as Synthetic Minority Oversampling Technique (SMOTE) to balance their training dataset. Imbalance in data could seriously affect the algorithm’s performance, in turn reducing the accuracy and stability of the models.

Thirdly, the majority of studies utilized univariate analysis for variable selection, which only considers the influence of a single factor on the outcome, potentially overlooking other impactful factors (27). This approach may result in the omission of crucial variables, leading to inaccurate prediction outcomes. Furthermore, univariate analysis does not account for interaction effects among predictive factors, which could introduce bias into the prediction results (28). Fourth, concerning model performance assessment and validation, the majority of studies primarily focused on metrics like AUC values and calibration. Some of them evaluated sensitivity, specificity, and accuracy of the models but failed to assess their clinical applicability. This indicates a need for further clinical validation of these existing models. It is important to note that none of the studies incorporated external validation, leading to a high risk of overfitting. Overfitting signifies that while the model performs well on the training dataset, its performance substantially drops on new, unseen data, thereby significantly reducing the accuracy and reliability of the predictions (29). Therefore, to address these limitations and enhance the predictive models for post-thrombectomy brain edema risk in stroke patients, future studies should include more diverse populations, implement rigorous data preprocessing, employ multivariate analysis, and conduct external validation.

### 4.4. Implications for future predictive models

#### 4.4.1. Data preprocessing

Among the included studies, only the research by Zeng addressed the issue of imbalanced data using the SMOTE method (15). This observation underscores that many researchers who are constructing predictive models for post-thrombectomy brain edema risk in stroke patients have not prioritized data preprocessing, which could lead to decreased predictive performance and reliability of the models. Data preprocessing is not only crucial for the accuracy of predictive models but also for ensuring data quality, reliability, and effectiveness. This process involves steps such as data cleaning, integration, and normalization (30). Properly preprocessed data can enhance model development by reducing noise and errors, thereby improving reliability and prediction accuracy. Furthermore, it can aid in the identification of issues within the dataset, enabling the formulation of more appropriate problem-solving strategies (31).

#### 4.4.2. Predictive factor selection

Among the five studies included in this review, only Jiang et al. utilized the Least Absolute Shrinkage and Selection Operator (LASSO) for predictive factor selection (14), while the remaining studies relied on univariate analysis. The latter approach may overlook important variables. As such, during the selection of predictive factors, a comprehensive screening of factors should be conducted in the preliminary stage using expert knowledge, correlation analysis, and meta-analysis. Further, during the model construction phase, the use of regularization methods such as LASSO and RIDGE can be beneficial for variable selection. This approach helps in reducing the risk of overfitting and in identifying variables that significantly contribute to the predictive target (32).

#### 4.4.3. External validation

The purpose of external validation is to assess the generalizability of predictive models, necessitating the use of datasets distinct from those used in the original study. Methods such as temporal, spatial, and domain validation can facilitate this process (33). Not only does this approach help mitigate the risk of overfitting, but it also serves as a crucial step in evaluating the model’s stability and applicability. Poor performance during external validation may even necessitate further adjustments to the model. Regrettably, the models included in this review lack external validation, which hampers their extrapolative potential and underscores the imperative for enhancements in subsequent research. While these models have exhibited robust theoretical performance, their effectiveness in real-world applications may be compromised by various factors, including patient characteristics and treatment methodologies. Consequently, future research endeavors should aim to test and fine-tune these models within clinical contexts to ensure their applicability and efficiency in practical scenarios.

Furthermore, in the future, when developing and validating prediction models, emphasis should be placed on including data from different regions and populations. This approach would help us better understand and address the impact of regional and demographic factors on predictive outcomes, thereby creating models with broader applicability and precision. During this process, factors such as patient age, gender, ethnicity, and lifestyle conditions that might influence the disease progression and prognosis should be taken into account. Through such methods, we can develop more comprehensive and accurate prediction models that are better suited for clinical practice.

## 5. Conclusion

In this study, through a meticulous, systematic search and selection process, we included a total of five articles, encompassing 10 predictive models for post-thrombectomy brain edema risk in stroke patients. Even though all models exhibited an AUC value exceeding 0.8, indicating satisfactory predictive performance, certain limitations still exist. Primarily, the majority of studies exhibited a high risk of bias and lacked external validation. In future research endeavors, it will be crucial to adhere strictly to PROBAST and TRIPOD guidelines and to consider diverse regions and populations for the development of predictive models with robust performance and high clinical applicability. Such models could serve as a valuable tool for healthcare professionals in the early identification of patients at high risk.

## Data availability statement

The original contributions presented in the study are included in the article/Supplementary material, further inquiries can be directed to the corresponding author.

## Author contributions

LL: Conceptualization, Formal analysis, Methodology, Software, Writing – original draft. C-yH: Methodology, Supervision, Writing – review & editing. J-xY: Methodology, Writing – original draft. S-tZ: Methodology, Writing – review & editing. JZ: Methodology, Writing – review & editing. YK: Data curation, Writing – review & editing. W-bC: Methodology, Writing – review & editing. YX: Formal analysis, Writing – review & editing.

## Conflict of interest

The authors declare that the research was conducted in the absence of any commercial or financial relationships that could be construed as a potential conflict of interest.

## Publisher’s note

All claims expressed in this article are solely those of the authors and do not necessarily represent those of their affiliated organizations, or those of the publisher, the editors and the reviewers. Any product that may be evaluated in this article, or claim that may be made by its manufacturer, is not guaranteed or endorsed by the publisher.
