# Supplementary material for: Prediction models for post-thrombectomy brain edema in patients with acute ischemic stroke: a systematic review and meta-analysis
Source: Front Neurol. 2023 Aug 31;14:1254090. doi: 10.3389/fneur.2023.1254090 (PMC10501604; doi:10.3389/fneur.2023.1254090)
Supplement: Supplementary file 1 [file Table_1.docx]

**Supplementary materials**

**Table S1. Search strategies**

1. **Search term**

**Ischemic stroke and large vessel occlusion**

#1 Ischemic Stroke[mesh term]

#2 Brain Ischemia[mesh term]

#3 Ischemic Encephalopathy OR Ischemic Encephalopathies OR Cerebral Ischemia* OR Ischemic Stroke* OR Cryptogenic Ischemic Stroke* OR Cryptogenic Stroke* OR Cryptogenic Embolism Stroke* OR Wake-up Stroke* OR Acute Ischemic Stroke*

#4 #1 OR #2 #3

#5 large vessel occlusion OR cerebrovascular occlusion OR endovascular thrombectomy OR mechanical thrombectomy

#6 #4 AND #5

**Prediction models**

"risk prediction model" OR "prediction model" OR "risk stratification"[Title/Abstract] OR "risk score" OR "risk assessment" OR "clinical prediction model" OR "model"[Title/Abstract] OR "predictor"

**Brain Edema**

"Brain Edema" OR "Cerebral Edema" OR "Intracranial Edema" OR "Brain Swelling*" OR "Cerebral Edemas, Vasogenic" OR "Cytotoxic Cerebral Edema"

1. **Retrieval：take pubmed as an example**

#1 Search: "Ischemic Stroke"[MeSH Terms]

#2 Search: "Brain Ischemia"[MeSH Terms]

#3 Search: "Ischemic Encephalopathy"[Title/Abstract] OR "Ischemic Encephalopathies"[Title/Abstract] OR "Cerebral Ischemia*"[Title/Abstract] OR "Ischemic Stroke*"[Title/Abstract] OR "Cryptogenic Ischemic Stroke*"[Title/Abstract] OR "Cryptogenic Stroke*"[Title/Abstract] OR "Cryptogenic Embolism Stroke*"[Title/Abstract] OR "Wake-up Stroke*"[Title/Abstract] OR "Acute Ischemic Stroke*"[Title/Abstract]

#4 Search: #1 OR #2 OR #3

#5 Search: "large vessel occlusion"[Title/Abstract] OR "cerebrovascular occlusion"[Title/Abstract] OR "endovascular thrombectomy"[Title/Abstract] OR "mechanical thrombectomy"[Title/Abstract]

#6 Search: "risk prediction model"[Title/Abstract] OR "prediction model"[Title/Abstract] OR "risk stratification"[Title/Abstract] OR "risk score"[Title/Abstract] OR "risk assessment"[Title/Abstract] OR "clinical prediction model"[Title/Abstract] OR "model"[Title/Abstract] OR "predictor"[Title/Abstract]

#7 Search: #4 AND #5 AND #6

#8 Search: "Brain Edema"[Mesh]

#9 Search: "Brain Edema"[Title/Abstract]  OR "Cerebral Edema"[Title/Abstract]  OR "Intracranial Edema"[Title/Abstract]  OR "Brain Swelling*"[Title/Abstract]  OR "Cerebral Edemas Vasogenic"[Title/Abstract]  OR "Cytotoxic Cerebral Edema"[Title/Abstract]

#10 #8 OR #9

#11 #7 AND #10

| **Table S2. Study parameters and brain edema criteria** | | | |  |
| --- | --- | --- | --- | --- |
| **Author** | **Inclusion Criteria** | **Target Population** | **Diagnosis Criteria for Brain Edema** | |
| Chen | NCCT scan within 24 hours after EVT | AIS patients, occlusion of ICA or MCA，underwent EVT | MLS defined as any deviation of the midline structures | |
| Du | 1. Age ≥ 18 years; 2. Time from stroke onset to puncture ≤ 8 hours; 3. Baseline NIHSS score ≥ 6, baseline Alberta Stroke Program Early CT score ≥ 6, and prestroke modified Rankin Scale score < 2; 4. The ICA or proximal segment (M1 or M2) of the MCA occlusion confirmed by preoperative imaging. | AIS patients, occlusion of ICA or MCA，underwent EVT | Based on imaging examination after 3-5 days, defined as midline shift >5mm or disappearance of basal cisterns | |
| Jiang | 1. Age ≥18 years; 2. Large-artery occlusion in the anterior circulation confirmed 3. By preoperative imaging; 4. Time from stroke onset to puncture ≤24 hours; 5. Baseline NIHSS score ≥6; 6. No primary intracranial hemorrhage detected in admission CT. | Occlusion of large vessels in the anterior cerebral circulation, underwent EVT | CT examination within 5 days after surgery, defined as midline shift >5mm at the location of pineal body or transparent septum | |
| Zeng | Confirmed AIS patients with anterior circulation large vessel occlusion who underwent MT , with successful recanalization (mTICI score 2b-3) | AIS patients, occlusion of ICA or MCA，underwent EVT | Diagnosis using NCCT scan within 24 hours | |
| Cheng | 1. Age ≥18 years; 2. Met the diagnostic criteria for acute ischemic stroke in the Chinese Guidelines for Diagnosis and Treatment of Acute Ischemic Stroke 2018; 3. The ICA or proximal segment (M1 or M2) of the MCA occlusion confirmed by preoperative imaging; 4. Preoperative AIbertaStroke Program Early CT Score ≥6; 5. NIHSS≥9; 6. The time from onset to femoral artery sheathing was not more than 6 h; 7. The immediate postoperative mTICI was not less than grade 2b; 8. The head was reviewed by CT or MRI within 72 h after the operation | AIS patients, occlusion of ICA or MCA，underwent EVT | 1. NIHSS score ≥4 or an increase of the consciousness evaluation part of the NIHSS score ≥1; 2. The range of the hypodense lesions was >50% of the supply area of the middle cerebral artery, and it was accompanied by signs of local brain edema | |

EVT: Endovascular Treatment; ICA: Internal Carotid Artery; MCA: Middle Cerebral Artery; MLS: Midline Shift; mTICI: Modified Thrombolysis in Cerebral Infarction

**Table S3. Quality assessment of each study by TRIPOD checklist**

| **Study** | **1 (D;V)** | **2 (D;V)** | **3a (D;V)** | **3b (D;V)** | **4a (D;V)** | **4b (D;V)** | **5a (D;V)** | **5b (D;V)** | **5c (D;V)** | **6a (D;V)** | **6b (D;V)** | **7a (D;V)** | **7b (D;V)** | **8(D;V)** | **9 (D;V)** | **10 a (D)** | **10b (D)** | **10c (V)** | **10d (D;V)** | **10e (V)** | **11 (D;V)** | **12 (V)** | **13a (D;V)** | **13b (D;V)** | **13c (V)** | **14a (D)** | **14b (D)** | **15a (D)** | **15b (D)** | **16 (D;V)** | **17 (V)** | **18 (D;V)** | **19a (V)** | **19b (D;V)** | **20 (D;V)** | **21 (D;V)** | **22 (D;V)** |
| --- | --- | --- | --- | --- | --- | --- | --- | --- | --- | --- | --- | --- | --- | --- | --- | --- | --- | --- | --- | --- | --- | --- | --- | --- | --- | --- | --- | --- | --- | --- | --- | --- | --- | --- | --- | --- | --- |
| chen | Y | Y | Y | Y | Y | Y | Y | Y | Y | Y | Y | Y | Y | N | Y | Y | Y | n.a | Y | n.a | N | n.a | Y | Y | n.a | Y | Y | N | N | Y | n.a | Y | n.a | Y | Y | Y | Y |
| Du | Y | Y | Y | Y | Y | Y | Y | Y | Y | Y | Y | Y | Y | N | Y | Y | Y | Y | Y | Y | Y | n.a | Y | Y | Y | Y | Y | Y | Y | Y | n.a | Y | Y | Y | Y | Y | Y |
| Jiang | Y | Y | Y | Y | Y | Y | Y | Y | Y | Y | n.a | Y | n.a | N | N | Y | Y | Y | N | n.a | Y | Y | Y | Y | Y | Y | N | n.a | N | N | n.a | Y | Y | Y | Y | Y | Y |
| zeng | Y | Y | Y | Y | Y | Y | Y | Y | Y | Y | Y | Y | Y | N | Y | Y | Y | n.a | Y | n.a | N | n.a | Y | Y | n.a | Y | N | N | N | Y | n.a | Y | n.a | Y | Y | Y | Y |
| cheng | Y | Y | Y | Y | Y | Y | Y | Y | Y | Y | Y | n.a | n.a | N | Y | n.a | Y | n.a | Y | n.a | n.a | n.a | Y | Y | n.a | Y | n.a | n.a | n.a | Y | n.a | Y | n.a | Y | Y | Y | Y |

Abbreviation: Y, reported; N, didn’t report; n.a, not assessed. D, items relevant to the development of a prediction model; V, items relevant to the validation of a prediction model; D;V, items relevant to both of the development and validation of a prediction model.
